# Thermal Environment and Behavior Analysis of Confined Cows in a Compost Barn

**DOI:** 10.3390/ani12172214

**Published:** 2022-08-28

**Authors:** Geovani Marques Laurindo, Gabriel Araújo e Silva Ferraz, Flavio Alves Damasceno, Joao Antônio Costa do Nascimento, Gabriel Henrique Ribeiro dos Santos, Patrícia Ferreira Ponciano Ferraz

**Affiliations:** 1 Agricultural Engineering Department, School of Engineering, Federal University of Lavras, Lavras 37200-000, MG, Brazil; 2 Engineering Department, School of Engineering, Federal University of Lavras, Lavras 37200-000, MG, Brazil; 3 Department of Agricultural Engineering, Federal University of Viçosa, Av. Peter Henry Rolfs, University Campus of Viçosa, Viçosa 36570-900, MG, Brazil

**Keywords:** YOLOv3, image analysis, dairy herd, animal behavior

## Abstract

**Simple Summary:**

The use of the compost barn system has increased in dairy farms as it provides greater well-being to animals, favoring productivity. Thus, studies related to the thermal environment and behavior are paramount to assessing animal welfare and optimizing management. The objective of this work was to characterize the thermal environment inside a compost barn and to evaluate the standing and lying behavior of cows through images covering the four seasons. Dry bulb temperature, dew point temperature, and relative humidity data were collected every 10 minutes during the analyzed days, calculating the temperature and humidity index (THI). Filming was performed inside the barn, which was analyzed visually and in an automated way to assess the behavior of these animals. For the automated analysis, an algorithm was developed using Artificial Intelligence tools, YOLOv3. It was observed that in the experimental period the highest mean values of THI were observed during the afternoon and autumn. The animals’ preference to lie down on the bed for most of the day was verified. Regarding the developed algorithm, it was observed that it could detect cow behavior (lying down or standing), concluding that artificial intelligence was successfully applied and can be recommended for such use.

**Abstract:**

The compost barn system has become popular in recent years for providing greater animal well-being and quality of life, favoring productivity and longevity. With the increase in the use of compost barn in dairy farms, studies related to the thermal environment and behavior are of paramount importance to assess the well-being of animals and improve management, if necessary. This work aimed to characterize the thermal environment inside a compost barn during the four seasons of a year and to evaluate the standing and lying behavior of the cows through images. The experiment was carried out during March (summer), June (autumn), August (winter), and November (spring). Dry bulb temperature (*t_db_*, °C), dew point temperature (*t_dp_*, °C), and relative humidity (RH,%) data were collected every 10 minutes during all analyzed days, and the temperature and humidity index (THI) was subsequently calculated. In order to analyze the behavior of the cows, filming of the barn interior was carried out during the evaluated days. Subsequently, these films were analyzed visually, and in an automated way to evaluate the behavior of these animals. For the automated analysis, an algorithm was developed using artificial intelligence tools, YOLOv3, so that the evaluation process could be automated and fast. It was observed that during the experimental period, the highest mean values of THI were observed during the afternoon and the autumn. The animals’ preference to lie down on the bed for most of the day was verified. It was observed that the algorithm was able to detect cow behavior (lying down or standing). It can be concluded that the behavior of the cows was defined, and the artificial intelligence was successfully applied and can be recommended for such use.

## 1. Introduction

The compost barn confinement system for dairy cattle has been sought by milk producers as an alternative to improve the production and the quality of the milk, thus achieving better financial return and greater comfort for the animals [1]. In Europe, the compost barn has been increasingly used, as it has already been widely used in Israel and in the United States, with many research data on this subject coming from Minnesota [2].

The compost barn comprises a bed area with free space and a feed aisle separated from the bed, usually by a wall or gap. The bedding is usually formed by a 20 to 25 cm high layer of wood shavings that absorbs water from the waste that decomposes together [3].

For animals to fully express their genetic potential, in addition to a balanced diet, adequate thermal conditions must be offered within the ambient temperature range in which the animal is not stressed by cold or heat, ensuring that it can have greater use of dietary energy, minimal physiological adjustment, normal body temperature and normal appetite [4].

The animal in a situation of discomfort tries to find ways to adapt to the environment through physiological, metabolic, and behavioral adjustments. These adaptations can result in greater energy expenditure and a reduction in their production potential [5].

Considering the requirements that animals impose in relation to temperature, humidity, environment, and nutrition, milk production in confinement systems appears as an alternative to mitigate environmental interference in their performance. However, the lack of knowledge about managing the facilities and the confined animals can harm their well-being. An environment that does not meet the requirements of the animals can cause several problems, such as reduced dry matter consumption, the occurrence of udder health, and fertility problems, among others, which will directly influence the animal’s productivity [6].

There are methodologies to classify the welfare of animals under the husbandry systems to which the animals are submitted. These methodologies can be divided into two categories: direct and indirect indicators. Indirect indicators are related to the environment to characterize the production and management system. Recording environmental parameters are generally easy, fast, and reliable. Direct indicators are related to the animal’s behavior, health, and physiology [7].

Cow positioning is a behavior of interest to the producer since research has already shown that it is beneficial for cattle to lie down for a certain time and that standing for long periods can mean some discomfort for the animal [8]. For the aid of behavior analysis, algorithms based on artificial intelligence models have been tested and improved to evaluate images of animals in their environment. Many researches have been developed using these computational tools, such as in a study about the behavior of cows by image analysis via visual method and by software to automatically detect dairy cow feeding and standing behaviors in free-stall barns [9]. Another study automatically evaluated dairy cows behavior (feeding or standing) using computer vision techniques. [10]. As it is possible to see, some papers related to computer vision techniques applied to dairy cows’ behaviors (such as standing and lying down) can be found for free-stall barns, but none can be found for compost barns.

Taking into account the factors discussed above, the present work seeks to characterize the thermal environment inside a compost barn and evaluate the behavior of dairy cattle using visual analysis and an analysis through the development of an algorithm based on artificial intelligence.

## 2. Materials and Methods

### 2.1. Confinement Environment

The experiment was carried out in a compost barn located in the municipality of Itaguara (Minas Gerais, Brazil), at 20°24′38.8″ S and 44°36′53.0″ W and climate classification Cwa (humid subtropical climate) according to Köppen classification [11].

**Figure 1:**
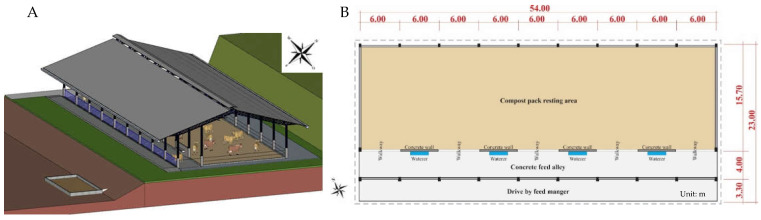
3D (**A**) and 2D (**B**) schematic drawing of the barn.

The evaluated barn has total dimensions of 23.0 × 54.0 m and a bed area of 15.7 × 54.0 m. The foot-right of the barn and the eaves are 4.8 m and 2.0 m, respectively (Figure 1). The corridor where the troughs and drinking fountains are located on the south-facing side of the installation. The feeding structure consists of a continuous trough running the length of the barn and four drinking troughs, between which five passages give access to the bedding area for the animals. The path of the animals to the milking waiting room is made on the opposite side of the feeding corridor, through three gates that give access to a corridor that connects the milking room. For mechanical ventilation inside the facility, two low-speed, high-volume fans were used (HVLS, BigFan^®^, 7.5 m diameter, 2.24 kW or 3.00 hp, and airflow of 650,000 m³.h^−1^).

### 2.2. Management and Herd

The property adopted the system of two milkings a day, the first at 5 am and the second at 3 pm. The herd of the property consisted of cows with blood grade 7/8 Girolando, composed of 51 cows, and an average daily milk yield was 18.4 L.

The material used to form the bed was the wood shavings in an initial layer of 30 cm, and the replacement was made according to the visual perception that the bed was excessively humid. In this way, a layer of 5 to 10 cm of wood shavings was added over the bed. The bed-turning operation was performed twice a day during the period when the animals were being milked, with an average duration of thirty minutes. The bed turning process used a hybrid implement (scarifier and rotary hoe) suitable for the turning work and coupled to a 50 hp tractor.

### 2.3. Environmental Parameters Evaluated in the Barn

Data were collected on eight different dates to cover the four seasons of the year: summer 03/05 and 03/06, autumn 06/18 and 06/19, winter 08/01 and 08/03, and spring 11/02 and 11/05 of the year 2021, During data collection, there wasn’t any interference in the herd’s routine, as most of the data collection was performed by datalogger sensors or film cameras. At each visit, data were collected regarding the thermal environment and videos of the animals in the facility.

The environmental variables evaluated, such as the dry bulb temperature (*t_db_*, °C), the dew point temperature (*t_dp_*, °C), and relative humidity (RH, %), were collected every 10 minutes for 24 h on all collection dates. To collect such variables, *t_db_*, *t_dp_*, and RH dataloggers (Instrutherm^®^, model HT-500) were used, with a precision for *t_db_* of ±0.1 °C, measurement range of −40 and 70 °C; RH with an accuracy of ±3%, and a measurement range from 0 to 100%), previously programmed to record data every 10 minutes at the height of 1.7 m from the bed in a container in which the animals could not access them. Five sensors/recorders were allocated inside the facility, and two sensors were used to collect data outside the barn.

The collected *t_db_*, and *t_dp_* data were converted into the temperature and humidity index (THI), according to the equation 1 developed by [12]:

(1) $$THI=t_{db}+0.36\times \left(t_{dp}\right)+41.5$$

where, *t*_*db*_ = dry bulb temperature (°C) and *t*_*dp*_ = dew point temperature (°C).

The environmental variables inside and outside the barn were analyzed using descriptive statistics using boxplot charts.

### 2.4. Evaluation of Animal Behavior by Images

In the compost barn installation, a security camera was installed (Intelbras Infra Hdcvi 720p Hd Vhd 1010b G4) that was used to collect images to evaluate the behavior of the animals during the evaluated period. Two screws next to the barn roof shears fixed the camera so that the viewing angle could encompass the bed area and the feeding aisle.

Eight visits were made to collect the videos, which underwent further analysis in two ways: visually and automatically through an algorithm developed for this analysis. Both methods’ analyses were performed on the same dates and times, as seen in Table 1. The behavior analyzed was for the animal to be in a lying or standing position.

#### 2.4.1. Visual Analysis of Behavior

The analysis to perform the visual count of the lying and standing animals consisted of observing the videos recorded at times listed in Table 1 and performing the visual count of the animals in each of the two behaviors, watching the video paused halfway through. These data were duly recorded in spreadsheets.

The visual analysis performed on the collected images took place during daytime periods due to the need for light to identify the animals’ positions in the barn. Night periods cause interference and difficulty for the observer to visually evaluate images. The variation of the solar declination changes along the year’s seasons, causing different hours of sun exposure each day, so the period of the video analysis suffered a slight variation according to the season.

#### 2.4.2. Automated Behavior Analysis Using Artificial Intelligence

To perform the automated analysis of the videos, a computer system based on artificial intelligence was developed to detect and count cows lying down or standing up. For this, the videos that appear in their dates and times in Table 1 were used.

Thus, the recognition of objects (animals) was performed from a network architecture of the third improvement of the YOLO algorithm (You Only Look Once) [13].

The main advantages of YOLOv3 are the 3 detection scales that reduce the original image in 16 × 16, 32 × 32 and 64 × 64 pixel grids, when considering an input image of 512 × 512 pixels. In addition, Darknet-53 was used as a feature extractor, an improved version of the Darknet-19 feature extractor used in YOLOv2, in which more convolutional layers and the use of residual blocks were introduced.

During YOLOv3 training, the grid cell in which the object center is inside is responsible for making the prediction. Each grid cell has three bounding boxes known as anchor boxes. Anchor boxes have their sizes previously selected based on database objects, facilitating the learning process. Thus, the network does not need to learn the geometric aspects of objects from scratch but rather only needs to adjust these anchor boxes so that the object can be located correctly.

The training images of the algorithm were obtained through the clipping (print). The first and last frames of 79 videos were extracted so a total of 158 images were taken. Thus, more videos were used than the times shown in Figure 1 to perform the algorithm training to increase its reliability. More samples were added through a process called data augmentation, in which horizontal mirroring was applied, that is, each image had its mirrored pair so the image database was duplicated from 158 to 316 images, thus being the final set of images used for training the algorithm. This technique is an artifice that artificially increases the number of images in a database using geometric transformations, such as mirroring (horizontally) the same images. Therefore, it helps to improve neural network learning during training, as a mirrored or rotated image is considered a new image, totally different from its original one, in the neural network.

**Figure 2:**
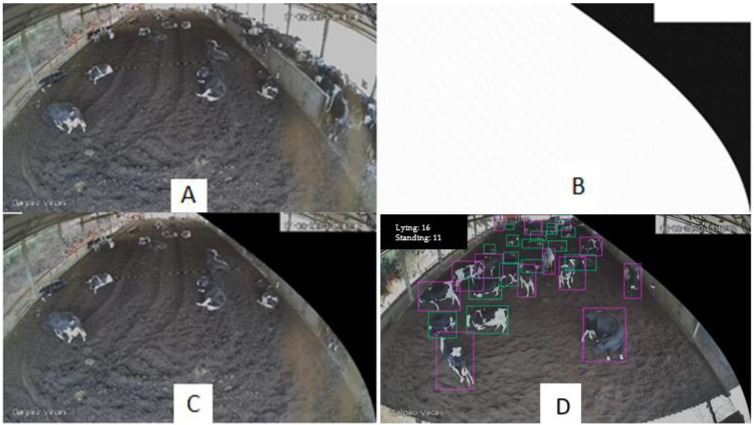
(**A**) Original image, (**B**) mask to remove the feeding aisle, (**C**) image used to train the algorithm and to count the animals, and (**D**) bounding boxes according to the animals’ behavior.

As the videos included the feeding corridor, and this location was not of interest in this study, a mask was placed over the frames (Figure 2), also including the training images, so that this corridor would not interfere with the counting of cows that were in the bed region.

The counting of animals was performed through a change in the code of the module used to design the bounding boxes of the Darknet framework [14], where two variables that account for objects found for each class of animal behavior (standing and lying down) were introduced (Figure 2D). The variables were updated every 30 frames (corresponding to 1 s), and were printed on the video resulting from the detection. To facilitate the comparative analysis between the visual and automated methods, some lines of code that record the number of objects of each class (lying down or standing) in a .csv file every 1 minute of video were also added.

#### 2.4.3. Validation of the Proposed Artificial Intelligence Model

With the information on the amount of each evaluated behavior duly tabulated, the behaviors were submitted to the transformation of values for percentages of animals in that behavior for both methods. Thus, scatter plots were made between the behaviors evaluated in a visual and automated way to compare the trend of values and grouping of data in the graphs. Behavior trend graphs were plotted for the visual and automated assessment so that the similarities of the curves in each method and in each season of the year could be observed side by side.

Time series analyses were also performed on the animal behavior data. These analyses were performed using the Orange Canvas 3.31.1 software [15].

Statistical analysis was performed using the Mann–Whitney test to compare the automated and visual methods. This test is equivalent to the nonparametric version of the t-test, which tests the equality of means. However, the Mann–Whitney test tests the equality of the medians, being used to test two independent samples [9]. This makes it possible to verify whether the similarity between methods was statistically significant or not. The count of lying and standing cows of each method was used to perform this comparison. The Mann–Whitney test was used because the data were obtained in different ways, so the test is important because it certifies whether or not the samples belong to the same population, and the objective of the work is that the counts were equal or minimally similar.

## 3. Results and Discussion

### 3.1. Evaluation of Environmental Variables

**Figure 3:**
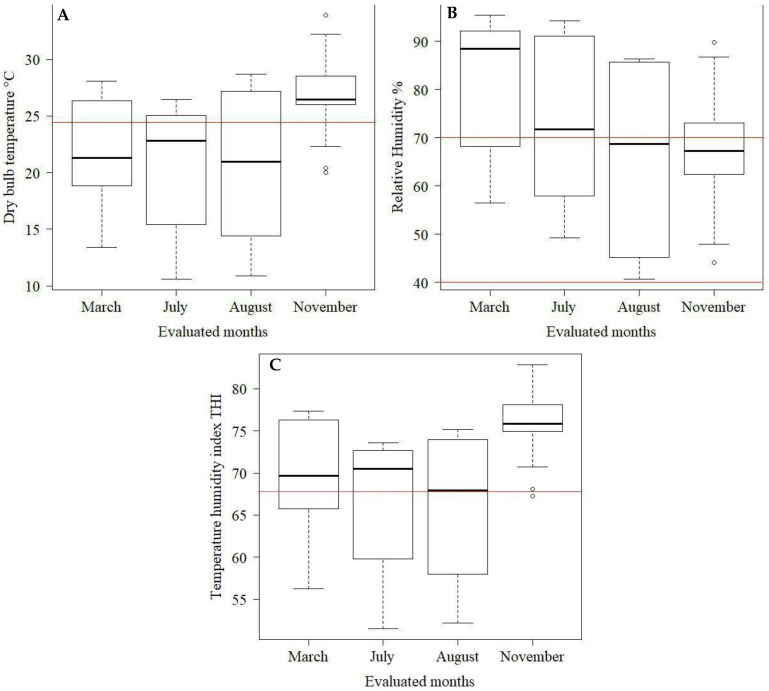
Box-plot of dry bulb temperature (**A**), relative humidity (**B**), and temperature humidity index (**C**) inside the barn during the experimental period. The red lines indicate the comfort values of the variables as described in the literature ([15,20,23]).

**Figure 4:**
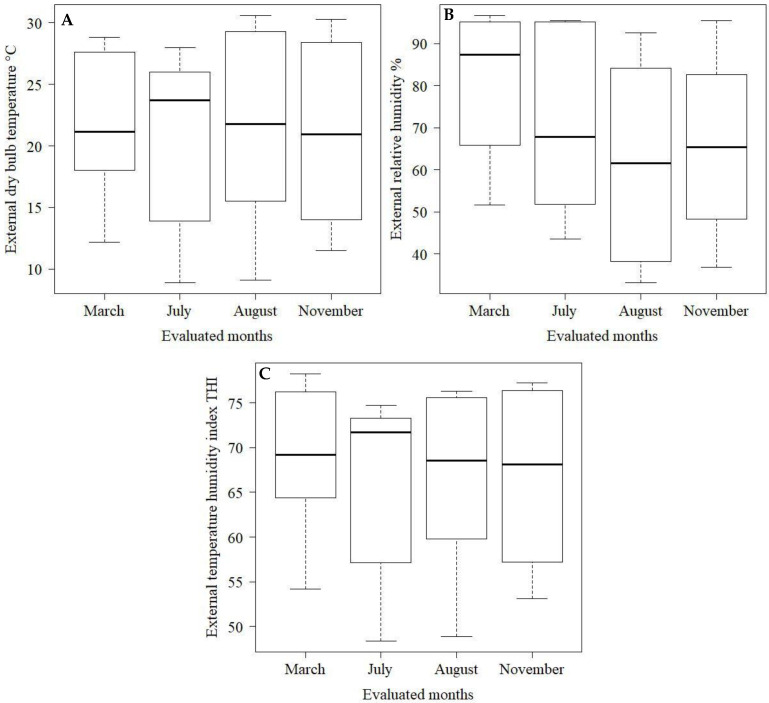
Box-plot of external dry bulb temperature (**A**), external relative humidity (**B**), and external temperature humidity index (**C**) during the experimental period.

Box-plot charts were used to assess the environmental variables inside (Figure 3) and outside (Figure 4) of the compost barn during the period evaluated. Observing Figure 3A, it is noted that during March (summer), June (autumn), and August (winter), the median values of *t_db_* (21.3; 22.8; 21.0 °C, respectively) were within the range of thermal comfort for dairy cows (5 to 25 °C) [16], which in the figure is indicated by the red lines. On the other hand, during the month of November (spring), the *t_db_* values internally (26.5 °C) were above the upper comfort limit (UCL) of 25 °C [17].

Heat stress is considered an important source of economic loss in livestock, causing effects on milk production, reproduction, calf mortality, and udder health [18]. Environmental conditions different from those considered comfortable for the animals can cause behavioral changes such as decreased food intake and increased water intake [19]. In addition, dairy cattle are very sensitive to high temperatures due to their high metabolic heat, due to milk production, and high rumen activity [20]. Research like this reinforces the need to keep the breeding environment for animals within the limits of thermoneutrality so that they can reach their maximum productivity and do not need to spend part of the ingested energy with physiological and behavioral adaptations in an attempt to maintain their body temperature.

Regarding RH (Figure 3B), throughout March and July, the median values were above comfort (88.4; 71.9, respectively), which ranges from 40 to 70% [21], and indicated in the figure by the red line. In a study that evaluated the thermal environment of a compost barn system, the author also observed higher RH values during periods of hot weather, which were higher than recommended in the literature [22]. High RH values can lead, in addition to increasing the probability of diseases in the teats and hooves, to the difficulty of dissipating heat through the air, which can cause losses of up to 10% in milk production [19].

As it involves a smaller number of variables, the THI is one of the simplest indices and has stood out for including the effects of *t_db_* and RH and, consequently, their effect on the thermal comfort of animals [23]. Most of the time, the THI (Figure 3C) presented median values higher than those considered adequate, which is 68 [24], for the rearing of lactating dairy cows, being 69.7; 70.5 and 75.9 for March (summer), June (autumn) and November (spring), respectively—only the month of August (winter) presented THI within what is considered as thermal comfort, with a median value of 67.9. This may be an indication that during the period evaluated, especially in November, which proved to be the most critical concerning the thermal environment, the architectural characteristics of the installation and the ventilation system adopted were not enough to maintain THI values within the recommended range, which consequently may be one of the causes of heat stress in animals.

Regarding the thermal conditions in the external environment of the installation, it is observed that the *t_db_* values (Figure 4A) in the external environment of the barn are within the comfort range for Dutch cattle (5 to 25 °C) [16]. Furthermore, it is observed that the internal and external *t_db_* are very similar for March (summer), June (autumn) and August (winter), with external temperatures in these months being at most 1 °C above External *t_db_* (Figure 3 and Figure 4). It is only noteworthy that in November (spring), the difference between the internal (26.5 °C) and external (20.9 °C) *t_db_* was greater than 5 °C. This may be an indication that the internal factors of the barn, such as the heat produced by the animals, the bedding, and the barn architecture itself, associated with a possible inefficiency of mechanical ventilation, may have been responsible for the high *t_db_* inside the barn, compared to the external environment.

Regarding the RH of the external air (Figure 4B), the same pattern of behavior observed in the internal environment is observed (Figure 3B). The month of March (summer) presented the highest RH than the other months evaluated. The median value of RH was 87.4% for March, while August (winter) had the lowest median value of RH (63%). This characteristic observed in the external environment is expected because the region’s climate is considered Cwa, which is characterized by a climate with a dry winter and a hot and humid summer [11].

In the evaluated months, the external THI (Figure 4C) remained between 68 in November and 71 in June, values very close to the upper comfort limit for dairy cows, which is 68 [23]. Despite the times of the year observed, the external THI showed little variability between the studied days.

### 3.2. Behavior Assessment through Images

**Figure 5:**
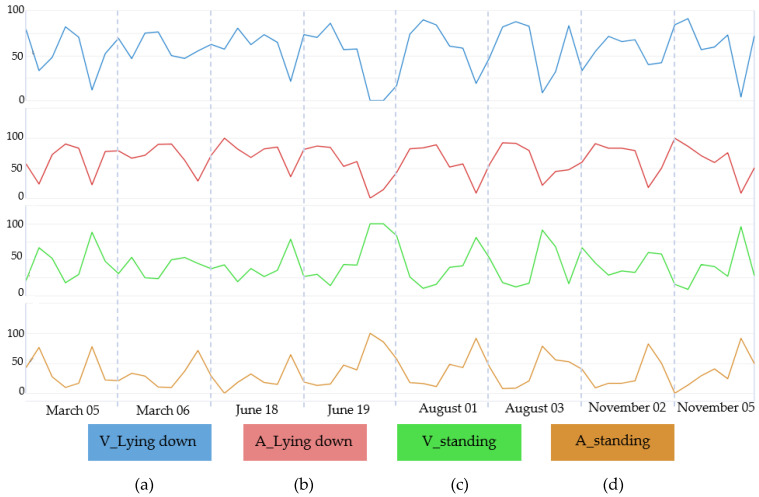
Time series of cow behavior over 1 year, with analysis performed visually (V) lying down (**a**), visually standing up (**c**), by the algorithm (A), lying down (**b**), and by the algorithm standing up (**d**).

To make it possible to evaluate the positioning behavior of the animals (standing and lying down) throughout the analyzed period, time series analysis graphs were made, considering the analyses performed visually and through an algorithm based on artificial intelligence (Figure 5). In Figure 5, it is possible to observe the behavior of the cows during the studied days and times (according to Table 1). Since it is a time series chart, there is no break between the days because this analysis made an interpolation in the data to build the time series model. Therefore, this graph also made it possible to observe the behavior of the cows in the compost barn throughout the seasons, visualizing the percentage of animals that presented the behavior lying down or standing up.

Visually comparing the format and trend of the graph lines, there is a similarity between the two types of analysis used (visual and algorithm), demonstrating that the tool used tends to be effective in its use for animal behavior analysis.

The lying position (Figure 5a,b) presents a grouping in the highest values, indicating that the cows preferred remaining lying down most of the time during the analyzed period. When the standing behavior was analyzed (Figure 5c,d), the data were grouped into smaller values of the percentage of time evaluated, thus demonstrating that the cattle prefer to remain in this position for a shorter period. According to studies, it is reported that dairy cows spend about 8 to 16 h a day lying down [25], and the time that the cow remains lying down at rest is positively associated with rumination [26]. It was observed in research that the act of ruminating and resting (laying down) are naturally done together, both activities start approximately 70 minutes after the end of feeding [22]. Such activity is directly associated with sleepiness, thus providing physiological rest for the animals. Rest, rumination and sleep have interconnections forming a set of important activities for the adjustment of the animals’ metabolic functions and immunity, consequently the influence on the health and well-being of the cows [27]. The daily rest time of each cow can be an indicator of animal welfare [28], in addition to measuring the comfort of the bed surface for rest and the housing system [29].

According to Figure 5, it is also observed that in the months of June (autumn) and August (winter), the cows spent the least time lying down and the longest time standing. In addition, in November (spring), they had the highest percentage of time lying down. When observing Figure 3, which characterizes the thermal environment inside the barn during the study period, it is observed that in June and August, the studied thermal variables were closer to what is considered as comfort. However, the month of November can be considered the most critical, presenting the highest median values of *t_db_* (26.45 °C) and THI (75.9). In the first study about the behavior of cows housed in the compost barn, the authors observed that resting time (lying down) and walking behavior in the compost barn were affected by THI inside the barn. Cows spent more time lying down and walked less when the THI was greater than 72 (12.7 h a day lying down and 71.6 steps/h) compared to when the THI was less than or equal to 72 (7.90 h lying down and 120.8 steps/h) [29]. The same behavior trend was observed in this experiment; cows remained lying down longer with higher THI values.

**Figure 6:**
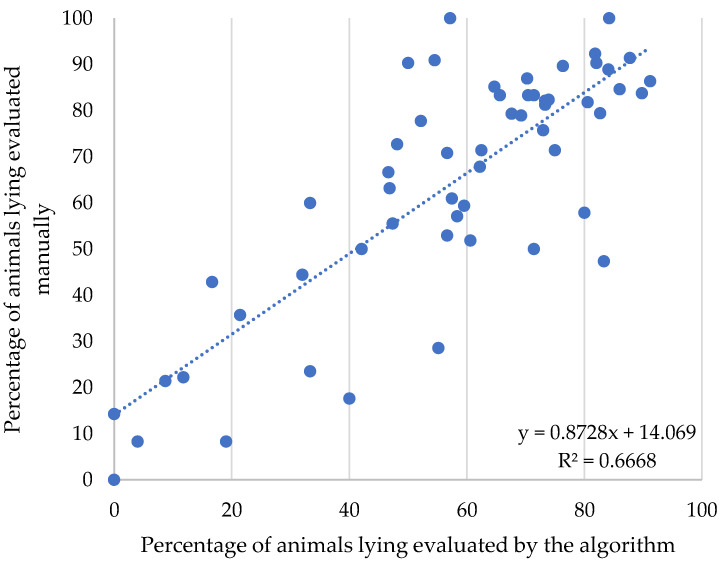
Dispersion for percentage of animals in manual count versus artificial intelligence count, for lying behavior.

**Figure 7:**
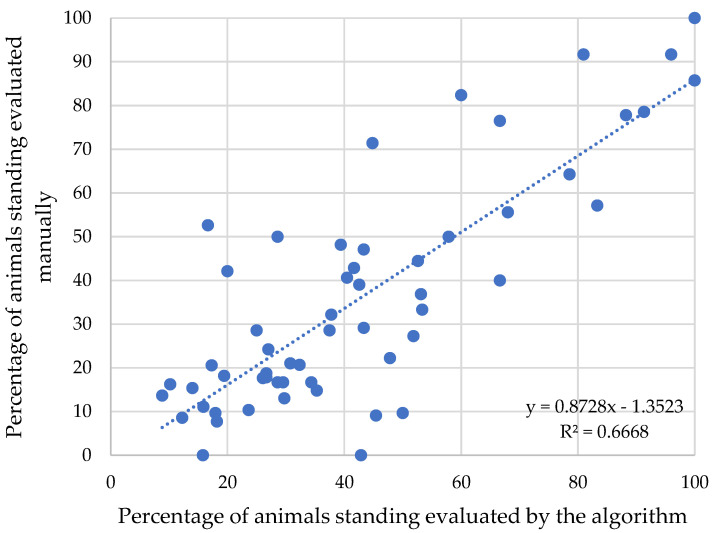
Dispersion for percentage of animals in manual counting versus artificial intelligence counting, for the standing behavior.

Figure 6 and Figure 7 indicate the correlation between the percentages of animals identified in lying behavior by visual counting versus counting via algorithms developed with artificial intelligence. The analysis presents R² coefficients of 66.68%, which means an adjustment of 66.68% of the automated method to the visual method. According to studies there is a high standard deviation between image analysis methods, and there is an indication of possible causes, such as the grouping of animals forming a single mass in the image and the spots on the coat that can confuse the image analyzed by the algorithm [30].

**Figure 8:**
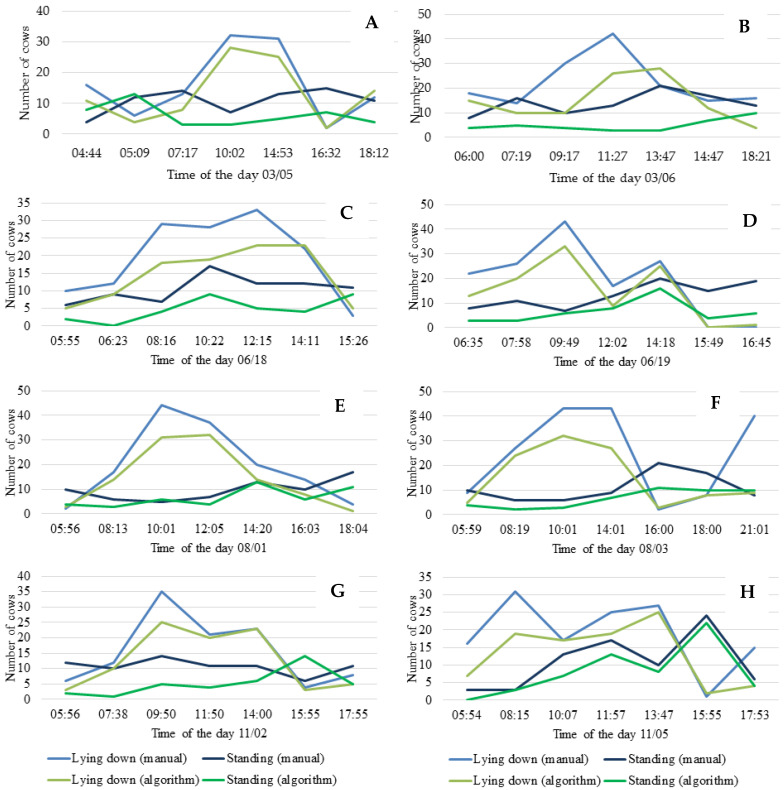
Behavior trend for cows lying down and standing with manual counting and counting by algorithm during summer on 03/05 (**A**) and 03/06 (**B**), in autumn on 06/18 (**C**) and 06/19 (**D**), in winter on 08/01 (**E**) and 08/03 (**F**), and in spring on 11/02 (**G**) and 11/05 (**H**).

The comparison of the evaluation of the algorithm developed with artificial intelligence with the visual evaluation can be done by analyzing the trend graphs grouped by season (Figure 8).

In Figure 8, the blue lines indicate the behavior trend of the cows evaluated by the visual method and the green lines indicate the evaluations made by the algorithm. Comparing the analysis methods, it is visually expressed that the lines of the graphs represent the same trend of behaviors, thus validating the efficiency of the algorithm developed in this research compared to the visual method for the different days analyzed.

Observing Figure 8A, which represents the summer (03/05), it can be seen that cows tend to lie down more between mid-morning and mid-afternoon, similar to studies that point out that cattle spend an average of 7 h and 40 min lying down and ruminating [31].

The automated evaluation of images performed by the algorithm showed greater assertiveness (Table 2) in summer (03/05 and 03/06) and in winter (08/01 and 08/03), presenting a lower and similar assertiveness in autumn and spring. This can be explained by the similar levels of solar radiation in the transition seasons (autumn and winter), which exerts a direct influence on the luminosity of the environment due to the angle of solar declination being similar in both seasons, a situation mentioned in another study, which also reported the difficulty in evaluating images at night due to low light [32].

When analyzing the position of the animals on the dates using the Mann–Whitney test (Table 3), it can be observed that there was a significant difference between the methods, despite the methods have shown the same trend of cattle positioning in the evaluated periods. In a study about the behavior of birds by image analysis via visual method and by software, the accuracy of the methodologies was evaluated by a Mann–Whitney test and were found values without significant difference between them, indicating that their analysis via artificial intelligence had similar precision to the visual method [9].

## 4. Conclusions

During the experimental period. it can be observed that the THI varied throughout the day and the year. The highest mean THI values were observed during the afternoon and autumn, Showing a slight level of thermal discomfort for the animals.

A visual analysis made it possible to define that cows in the compost barn spend most of their time lying down. It was possible to develop an algorithm based on artificial intelligence to identify the cows’ behavior (standing and lying down) inside the compost barn installation. The evaluation through this algorithm was effective when compared with the manual visualization method, presenting good levels of assertiveness. Therefore, artificial intelligence was successfully applied and can be recommended to analyze cows’ behavior inside a compost barn.

## Figures and Tables

**Table 1:** Date and time of filming to perform behavior analysis in a visual and automated way.

Summer				Autumn			
Day	Time	Day	Time	Day	Time	Day	Time
03/05/2021	04:44	03/06/2021	06:00	06/18/2021	05:55	06/19/2021	06:35
03/05/2021	05:09	03/06/2021	07:19	06/18/2021	06:23	06/19/2021	07:58
03/05/2021	07:17	03/06/2021	09:17	06/18/2021	08:16	06/19/2021	09:49
03/05/2021	10:02	03/06/2021	11:27	06/18/2021	10:22	06/19/2021	12:02
03/05/2021	14:53	03/06/2021	13:47	06/18/2021	12:15	06/19/2021	14:18
03/05/2021	16:32	03/06/2021	14:47	06/18/2021	14:11	06/19/2021	15:49
03/05/2021	18:12	03/06/2021	18:21	06/18/2021	15:26	06/19/2021	16:45
**Winter**				**Spring**			
**Day**	**Time**	**Day**	**Time**	**Day**	**Time**	**Day**	**Time**
08/01/2021	05:56	08/03/2021	05:59	11/02/2021	05:56	11/05/2021	05:54
08/01/2021	08:13	08/03/2021	08:19	11/02/2021	07:38	11/05/2021	08:15
08/01/2021	10:01	08/03/2021	10:01	11/02/2021	09:50	11/05/2021	10:07
08/01/2021	12:05	08/03/2021	14:01	11/02/2021	11:50	11/05/2021	11:57
08/01/2021	14:20	08/03/2021	16:00	11/02/2021	14:00	11/05/2021	13:47
08/01/2021	16:03	08/03/2021	18:00	11/02/2021	15:55	11/05/2021	15:55
08/01/2021	18:04	08/03/2021	21:01	11/02/2021	17:55	11/05/2021	17:53

**Table 2:** Percentage of correct counting by software over manual counting.

Scheme 73	Percentage
Summer	73%
Autumn	54%
Winter	69%
Spring	55%

**Table 3:** Measures of central tendency (median) of the data obtained through the computational method and the visual method, referring to the lying and standing positions.

Date	Visual Method		Algorithm Method	
	Lying	Standing	Lying	Standing
March/05	16.00 ^a^	10.85 ^b^	13.14 ^c^	6.14 ^d^
March/06	22.28 ^a^	14.00 ^b^	15.00 ^c^	5.14 ^d^
June/18	19.57 ^a^	10.57 ^b^	14.57 ^c^	4.71 ^d^
June/19	19.28 ^a^	13.28 ^b^	14.42 ^c^	6.57 ^d^
August/01	19.71 ^a^	9.71 ^b^	14.71 ^c^	6.71 ^d^
August/03	24.57 ^a^	11.00 ^b^	15.42 ^c^	6.71 ^d^
November/02	15.57 ^a^	10.71 ^b^	12.71 ^c^	5.28 ^d^
November/05	18.85 ^a^	10.85 ^b^	13.28 ^c^	8.14 ^d^

Medians followed by the same letter horizontally do not differ significantly by the Mann–Whitney test at 1% significance.

## Data Availability

The data presented in this study are available on request from the corresponding author.

## References

[1] De Oliveira A.P., da Silva O.P.R., da S. Bandeira N.V., da silva D.F., Silva J.A., Pinheiro S.M.G. (2014). Rendimento de maxixe em solo arenoso em função de doses de esterco bovino e biofertilizante. Rev. Bras. Eng. Agrícola Ambient..

[2] Ofner-Schröck E., Zähner M., Huber G., Guldimann K., Guggenberger T., Gasteiner J. (2015). Compost Barns for Dairy Cows—Aspects of Animal Welfare. Open J. Anim. Sci..

[3] Endres M.I., Barberg A. (2006). Compost Barns: What Have We Learned So Far?. Minn. Dairy Health Conf..

[4] Baccari F. (2001). Manejo ambiental para produção de leite em clima quentes. Congresso Brasileiro de Biometeorologia.

[5] Baêta F.C., Souza C.F. (2010). Ambiência em Edificações Rurais Conforto Animal.

[6] Bach A., Giménez A., Juaristi J.L., Ahedo J. (2007). Effects of physical form of a starter for dairy replacement calves on feed intake and performance. J. Dairy Sci..

[7] Calamari L., Bertoni G. (2009). Model to evaluate welfare in dairy cow farms. Ital. J. Anim. Sci..

[8] Scheibe K., Gromann C. (2006). Application testing of a new three-dimensional acceleration measuring system with wireless data transfer (WAS) for behavior analysis. Behav. Res. Methods.

[9] Porto S.M., Arcidiacono C., Anguzza U., Cascone G. (2015). The automatic detection of dairy cow feeding and standing behaviours in free-stall barns by a computer vision-based system. Biosyst. Eng..

[10] Porto S.M., Arcidiacono C., Anguzza U., Cascone G. (2013). A computer vision-based system for the automatic detection of lying behaviour of dairy cows in free-stall barns. Biosyst. Eng..

[11] Martins F.B., Gonzaga G., Dos Santos D.F., Reboita M.S. (2018). Classificação Climática de Köppen e de Thornthwaite para Minas Gerais: Cenário Atual e Projeções Futuras. Rev. Bras. Climatol..

[12] Thom E.C. (1958). Cooling Degrees—Days Air Conditioning, Heating, and Ventilating. Trans. ASAE.

[13] Redmon J., Divvala S., Girshick R., Farhadi A. You Only Look Once: Unified, Real-Time Object Detection. Proceedings of the IEEE Conference on Computer Vision and Pattern Recognition (CVPR).

[14] Redmon J. Darknet: Open Source Neural Networks in c. http://pjreddie.com/darknet/.

[15] Demsar J., Curk T., Erjavec A., Gorup C., Hocevar T., Milutinovič M., Možina M., Polajnar M., Toplak M., Staric A. (2013). Orange: Data Mining Toolbox in Python. J. Mach. Learn. Res..

[16] Roenfeldt S. (1998). You can’t afford to ignore heat stress. Dairy Manag..

[17] Pilatti J.A. (2017). O comportamento Diurno e o Bem-Estar de Vacas em Sistema de Confinamento Compost Barn. Master’s Thesis.

[18] Ricci G.D., Orsi A.M., Domingues P.F. (2013). Estresse calórico e suas interferências no ciclo de produção de vacas de leite: Revisão. Veterinária E Zootec..

[19] Head H.H. (1995). Management of dairy cattle in tropical and subtropical environments: Improving production and reproduction. Anais do 1o Congresso Brasileiro de Biometeorologia.

[20] Collier R.J., Hall L.W., Rungruang S., Zimbleman R.B. (2012). Quantifying Heat Stress and Its Impact on Metabolism and Performance.

[21] Dalcin V.C. (2013). Parâmetros Fisiológicos em Bovinos Leiteiros Submetidos ao Estresse Térmico.

[22] Pereira M.R. (2017). Avaliação do Comportamento e do Bem-Estar de Vacas Criadas em Sistema Compost Barn em Condições Tropicais. Master’s Thesis.

[23] Ponciano P.F., Junior T.Y., Lima R.R.D., Schiassi L., Teixeira V.H. (2012). Adjust of regression models to estimate the rectal temperature of broilers for the first 14 days of life. Eng. Agrícola.

[24] Rosenberg L.J., Biad B.L., Verns S.B. (1983). Human and animal biometeorology. Microclimate, the Biological Environment.

[25] Haley A.M., de Passillé J.R. (2001). Assessing cow comfort: Effects of two floor types and two tie stall designs on the behaviour of lactating dairy cows. Appl. Anim. Behav. Sci..

[26] Schirmann K., Chapinal N., Weary D.M., Heuwieser W., Von Keyserlingk M.A.G. (2012). Rumination and its relationship to feeding and lying behavior in Holstein dairy cows. J. Dairy Sci..

[27] Smutný L., Smutná Š., Kindlová J., Šoch M., Škeřík V., Zábranský L. (2013). The Usage of Information Technology for Evaluation of Animal Welfare. Anim. Sci. Biotechnol..

[28] Drissler M., Gaworski M., Tucker C.B., Weary D.M. (2005). Free-stall maintenance: Effects on lying behavior of dairy cattle. J. Dairy Sci..

[29] Endres M.I., Barberg A.E. (2007). Behavior of Dairy Cows in an Alternative Bedded-Pack Housing System. J. Dairy Sci..

[30] Souza S.R.L., Nääs I.A., Moura D.J. (2011). Análise de imagens para a caracterização das atividades de vacas leiteiras dentro do galpão de confinamento. Eng. Agrícola Jaboticabal.

[31] Grant R.J., Dann H.M. (2015). Biological Importance of Rumination and Its Use on-farm.

[32] Veit H.M., Salman A.K.D., Cruz P.G., Souza E.C., Schmitt E. (2018). Bioacústica como método de avaliação do comportamento em pastejo de novilhas Girolando. Arq. Bras. Med. Veterinária Zootec..

